# Methodological Challenges in Assessing the Environmental Status of a Marine Ecosystem: Case Study of the Baltic Sea

**DOI:** 10.1371/journal.pone.0019231

**Published:** 2011-04-29

**Authors:** Henn Ojaveer, Margit Eero

**Affiliations:** 1 Estonian Marine Institute, University of Tartu, Pärnu, Estonia; 2 National Institute of Aquatic Resources, Technical University of Denmark, Charlottenlund, Denmark; Institute of Marine Research, Norway

## Abstract

Assessments of the environmental status of marine ecosystems are increasingly needed to inform management decisions and regulate human pressures to meet the objectives of environmental policies. This paper addresses some generic methodological challenges and related uncertainties involved in marine ecosystem assessment, using the central Baltic Sea as a case study. The objectives of good environmental status of the Baltic Sea are largely focusing on biodiversity, eutrophication and hazardous substances. In this paper, we conduct comparative evaluations of the status of these three segments, by applying different methodological approaches. Our analyses indicate that the assessment results are sensitive to a selection of indicators for ecological quality objectives that are affected by a broad spectrum of human activities and natural processes (biodiversity), less so for objectives that are influenced by a relatively narrow array of drivers (eutrophications, hazardous substances). The choice of indicator aggregation rule appeared to be of essential importance for assessment results for all three segments, whereas the hierarchical structure of indicators had only a minor influence. Trend-based assessment was shown to be a useful supplement to reference-based evaluation, being independent of the problems related to defining reference values and indicator aggregation methodologies. Results of this study will help in setting priorities for future efforts to improve environmental assessments in the Baltic Sea and elsewhere, and to ensure the transparency of the assessment procedure.

## Introduction

An ecosystem approach to management (EAM) of the marine environment with the primary goal to achieve sustainable use of its goods and services is included in several policy documents at global and regional levels (e.g., [1], [2]). Such an approach to management requires, among other things, integrated ecosystem assessments to inform management decisions and regulate human pressures [3], [4], [5]. Indicators are generally accepted as tools for evaluating the status of marine environments in relation to management targets or thresholds [2], [6]. Despite the crucial role of indicators in helping to safeguard and manage environmental values, indicator-based ecosystem assessments entail challenges. A large part of related research has dealt with the characteristics of a good indicator [7], [8], [9]. Deriving appropriate thresholds is usually even more challenging than developing the indicators themselves [5], [10]. Substantially less research has focused on the sensitivity of the assessment results to the choice of indicators and the assessment methodologies applied, and on related uncertainties in overall evaluation of environmental status of an ecosystem.

In the Baltic Sea, recent policy-oriented actions toward regional application of an ecosystem approach to management of marine ecosystems are among the strongest in Europe. The Baltic Marine Environment Protection Commission (Helsinki Commission, HELCOM) has adopted the Baltic Sea Action Plan (BSAP) to help the Baltic Sea achieve “good environmental status by 2021” [11]. The four strategic goals defined in BSAP are “Baltic Sea unaffected by eutrophication,” “Baltic Sea with life undisturbed by hazardous substances,” and “Maritime activities carried out in an environmentally friendly way,” all of which should lead to a “Favourable conservation status of biodiversity.”

Recent thematic assessments of two of the BSAP strategic goals (biodiversity and eutrophication) [12], [13] provide systematic overviews both on the available datasets and on the dynamics of various ecosystem components related to these sectorial topics. Initial holistic assessment of the ecosystem health of the Baltic Sea [14] has evaluated progress of the implementation of BSAP, though the assessment is considered preliminary and requires further improvement both in methodology and in a knowledge base [14]. Further, the methodologies used in these assessments are neither entirely unified nor fully transparent.

In addition to the activities led by HELCOM, integrated ecosystem assessments in several sub-areas of the Baltic Sea have recently been carried out by ICES [15]. These analyses have used more sophisticated and unified methodology, however, have mainly focused on identifying and characterizing the ecological regime shifts (e.g., [16], [17]), with only limited direct implications for the policy and governance regarding the BSAP [18].

In addition to the datasets used in these systematic assessments, large amount of monitoring data is regularly gathered by HELCOM, and published in the form of Indicator Fact Sheets. This knowledge base, containing ecosystem information from hydrography to the upper trophic levels, has never been analysed in a systematic way, nor has the performance of these indicators been evaluated in relation to the agreed goals of BSAP. Further, reference levels for indicators corresponding to thesepolicy goals, are largely not defined as yet. Thus, indicator-based ecosystem assessment (and management) of the Baltic Sea is facing a number of future challenges.

In this paper, we use the central Baltic Sea as a case study to investigate some criticial methodological aspects involved in a holistic ecosystem assessment. Based on the best available scientific knowledge, we first define thresholds for all available indicators. We then conduct ecosystem assessments by the three major BSAP strategic goals (biodiversity, eutrophiction and hazardous substances), applying different methodological approaches. We particularly focus on i) the sensitivity of assessment results to different indicator aggregation rules, and ii) the trend analyses as an alternative or supplement to an evaluation in relation to indicator thresholds. Our aim is to identify which conclusions concerning the status of the three BSAP segments are robust to the selection of indicators and assessment methodologies, and where the methodological choices are critical for the outcome of the assessment. Our analyses can, thus, help establish priorities for future efforts to improve assessment of environmental status and can help to enhance the transparency of the assessment procedure in the Baltic Sea and elsewhere.

## Materials and Methods

### General description of the study area

The Baltic Sea is epicontinental and semienclosed sea with total volume of about 22×10^3^ km^3^ and the mean depth of 60 m. It is situated in the transition area of Atlantic marine and Eurasian continental climate systems. The Baltic Sea is characterized by a strong southwest-northeast salinity gradient (with saline water inflow from southwest) and north-south temperature gradient. It is composed of three macroregions – the Transition Area, Large Gulfs and the Baltic Proper [19], the latter being the focus area of this paper.

The Baltic Sea was formed after the last glaciation with the contemporary “ecological age” of about 8,000 years. Large catchment area with about 85 million inhabitants and long water residence time (25–35 years) [20] make the Baltic Sea especially vulnerable to a variety of human activities. The most important human activities influencing the environmental status of the Baltic Sea are pollution, maritime shipping, fisheries, nutrient input [21], and recently also increasing energy production and pipelines.

### Objectives and indicators

This paper focuses on three overarching strategic goals of the HELCOM BSAP, i.e., biodiversity, eutrophication and hazardous substances, and the specific agreed ecological objectives related to each goal [11]. As a first step, we compiled all available datasets that could be used as indicators of the status of the central Baltic Sea in relation to these objectives. We used in totoal 110 state indicators, 30 of which were related to biodiversity, 25 to eutrophication and 55 to hazardous substances. These data were supplemented by 32 indicators of human pressures. For the purpose of this paper, no prior selection of indicators was made, but all available relevant datasets for which thresholds (see below) could be defined, were included in the analyses.

Detailed descriptions of each indicator, time period of coverage, and data sources are provided in Tables S1, S2, S3, S4 and Text S1.

### Indicator thresholds

For each state indicator time series, we defined two of the three thresholds, that is, a value representing reference (target), acceptable, or bad conditions. The defined values with detailed justifications are provided in Table S2 and Text S1. The basic criteria used for defining indicator thresholds are described below.

“Reference” conditions were defined as:

- The level which can be considered natural. This was based either on long-term data extending back to historical time-periods when human impact was low or on the information from other areas where particular issue is not of major concern.
- The level, which corresponds to a condition where recovery of an organism group from a long-lasting and severe human pressure has taken place.
- Desirable level, where this is straightforward to define (e.g., no presence of organic pollutants that naturally do not occur in the marine environment or indicator levels that correspond to normal reproduction of marine organisms).
- Observed conditions if these have been more positive than the levels, which have been defined as acceptable in some EU or national regulation.

“Acceptable” conditions were defined:

- The level set by EU or national regulations (e.g., concentration of residual contaminants in fish).
- Expert-opinion based deviation from the reference conditions.
- The level below which the situation is considered to become critical (e.g., requires extra management action, critical for reproduction of marine organisms etc.).

Thresholds corresponding to “bad” conditions were defined:

- The most negative situation observed during the available time-series, after which conditions have improved.
- The level corresponding to a reproduction failure of some marine organisms.

### Indicator transformation

Using the defined thresholds, the individual indicator time series were transformed to common units on a scale from −1 to 1, which is a standard procedure in knowledge-based systems [22]. For every indicator, the transformation returns a value of −1 at a threshold that corresponds to “bad” conditions (*X*_−1_) and a value of 1 at a threshold that corresponds to “reference” conditions (*X*_1_). The threshold corresponding to “acceptable” conditions (*X*_0_) returns a transformation value of zero. The transformed equivalents for original values between the thresholds were calculated assuming a linear relationship. For indicators for which thresholds for *X*_0_ and *X*_1_ were defined, the linear relationship was subsequently extended to obtain negative transformed values; the value corresponding to −1 thus became the same distance from *X*_0_ as the distance of *X*_1_ from *X*_0_, determined by the defined thresholds. Similarly, when thresholds for *X*_−1_ and *X*_0_ where defined, the linear relationship was extended to identify *X*_1_. When thresholds corresponding to *X*_−1_ and *X*_1_ were defined, the whole range of intermediate values between −1 and 1 were derived directly from the linear relationship between the original and transformed values. The values outside the range of −1 to 1 in the transformed scale were set to −1 or 1, respectively, before aggregation of indicators.

Potential nonlinear relationships between the indicator values and the corresponding status of an ecosystem could be expected. However, the shape of these functions is seldom known and would likely be indicator-specific. In a holistic ecosystem assessment, involving a large number of indicators, consistent treatment of all indicators may be preferred. Linear approximation for transforming values between the thresholds is commonly used (e.g., [22]) and this approach was also adopted here.

To visualize long-term changes in the status of different components of the ecosystem, the transformed continuous scale (from −1 to 1) was converted into a five-point scale, each of the resulting five categories representing an interval on a continuous scale.

### Indicator aggregation

A holistic ecosystem assessment requires integration of information from a large number of individual indicators into an overall evaluation of the state of the ecosystem. Different methodologies can be applied for aggregating indicators, which vary, amongst others, in the way the outliers influence the aggregate value. The choice of indicator aggregation methodology can therefore be essentially important to the overall outcome of the assessment. In this paper, we have applied six different aggregation procedures of transformed indicators to evaluate the state of the ecosystem. Each aggregation procedure resulted in a single value related to each objective and further to each overarching goal. The aggregation rules applied were:

- Hierarchical mean (see Tables S3 for the structure of aggregation), where at each step of aggregation, the transformed indicator values were averaged.
- Hierarchical median (at each step of aggregation, the median of transformed indicator values was applied).
- Hierarchical fuzzy AND (at each step of aggregation, the fuzzy AND rule [22] was applied; see also below).
- Similar to (i–iii), but applying flat, i.e. non-hieracrhical aggregation instead of hierarchical one, for mean, median and fuzzy AND rules. Flat aggregation implies that all indicators related to a particular objective were aggregated at the same level, without prior groupings.

The fuzzy AND [22] is calculated as
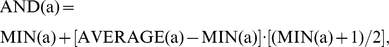
where

MIN (a) is the minimum value of input variables

and

AVERAGE (a) is the average value of input variables.

Fuzzy AND is a conservative way of aggregation and gives an aggregate close to the most negative value in an indicator suite.

### Analyses of trends

We analyzed trends in individual indicator time series to obtain information on the current situation of the ecosystem, independent of the challenges (such as definition of reference levels and aggregation of indicators) related to the assessment described above. Trends were estimated from linear regression using the i) five and ii) ten most recent data points in an indicator time series. The significant slope (*p*<0.1) was used as a criterion for identifying either a positive or a negative trend.

### Changes in human pressures

For pressure indicators, we did not attempt to define thresholds corresponding to target or acceptable levels, due to lack of relevant scientific basis. Instead, we show temporal developments in selected human pressures, both as trends in recent years and as longer term developments. Recent trends were estimated from linear regression, using the five most recent data points in a time-series. Long-term changes in pressures were shown relative to the highest level observed in the available time series. Some pressures presented in this paper are aggregates of several indicators (Tables S1 and S4).

## Results

### Current state of the ecosystem applying different indicator aggregation rules

**Figure 1:**
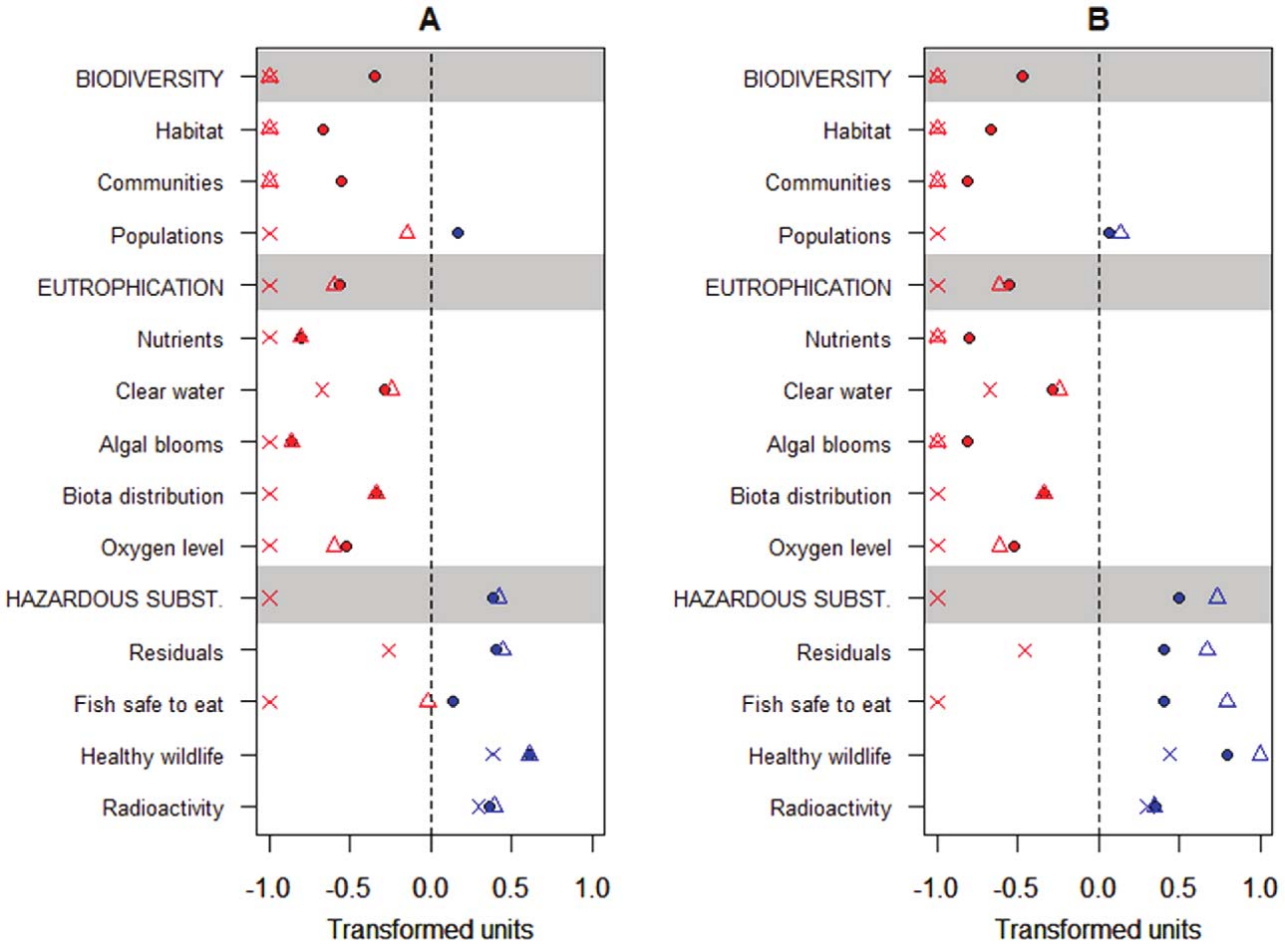
Current status of the central Baltic Sea ecosystem by overarching strategic goals and ecological objectives [**11**]. The negative values (marked in red) represent below acceptable or neutral status (zero-level) and positive values (marked in blue) represent the status above neutral. The different values on panels A and B are calculated based on (i) average of respective indicators (filled circles), (ii) median values of indicators (triangles), and (iii) applying the fuzzy AND rule for indicator aggregation (crosses). Panel A: indicators are aggregated hierarchically; panel B: flat (i.e., non-hierarchical) aggregation is applied.

The assessment of current environmental status based on average values of indicators suggests that, of the three BSAP strategic goals, the goal related to hazardous substances is currently being met at an acceptable level, as all affiliated objectives received positive scores in the evaluation (Figure 1). In contrast, all ecological quality objectives related to eutrophication received negative scores (Figure 1). Similarily, the overall status of biodiversity was evaluated as negative, with habitats and communities scored in poor condition, whereas some objectives (populations) received slightly positive values (Figure 1).

**Figure 2:**
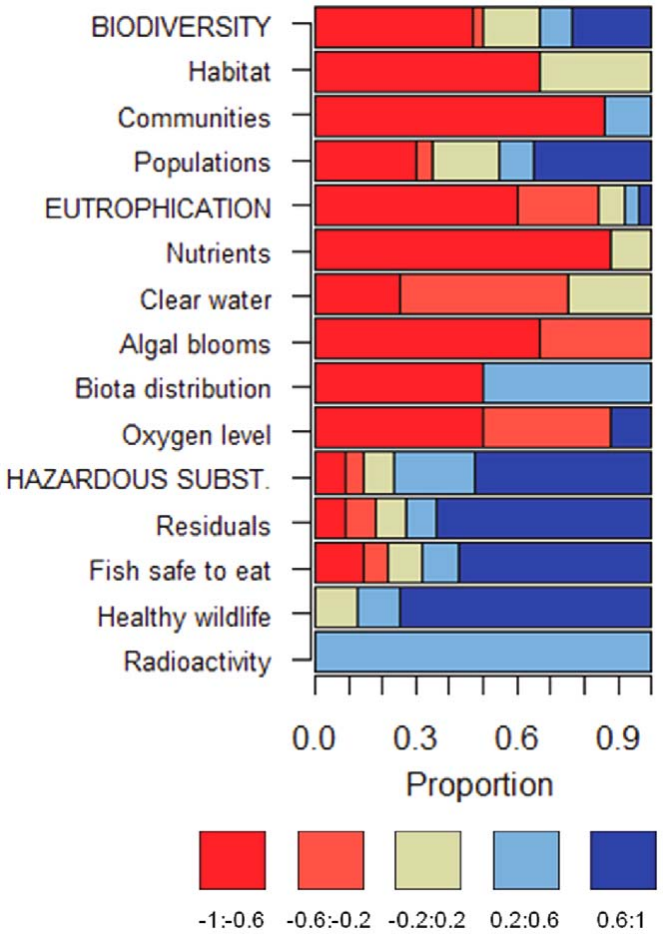
Current states of individual indicators related to a given ecological objective and overarching strategic goal. The current states are shown as a proportional distribution among five categories (obtained by dividing the scale from −1 to 1 into five intervals differentiated by colors).

For eutrophication and hazardous substances, the assessment results based on medians were very similar to these applying the average values of indicators. However, substantial differences were evident between average and median based assessments for biodiversity. The assessment based on medians resulted in most negative overall score, similar to the asssement applying the conservative fuzzy AND rule. This is due to only a few indicator datasets being available for habitats and communities (Table S3) with most of them showing strongly negative values (Figure 2).

Application of the conservative fuzzy AND rule resulted in the most negative scores in the assessment scale for most of the objectives by all three overarching strategic goals of BSAP (Figure 1). This assessment result is due to a fact that for nearly all of the ecological quality objectives, the current status of at least one affiliated indicator was strongly negative. The strong negativity of these indicators drove the outcome of an assessment when applying the conservative aggregation rule and resulted in an evaluation score close to the most negative value in an indicator suite.

The level of hierarchy applied in the aggregation of indicators appeared not to have a substantial influence on the outcome of the assessment. The results from non-hierarchical aggregation were generally similar to hierarchical assessment, regardless of the aggregation rule applied (i.e., average, median or fuzzy AND). However, some differences were apparent. For objectives where positive indicator scores dominated over negative ones, application of flat aggregation method resulted in a more positive evaluation compared to the hierarchical one. This is most evident for objectives related to hazardous substances (Figure 1,2). However, the opposite is apparent for biodiversity, where the dominant negative indicator values resulted in slightly more negative overall evaluation when applyingflat aggregation method, compared to hierarchical aggregation.

### Long-term changes in state and pressures

**Figure 3:**
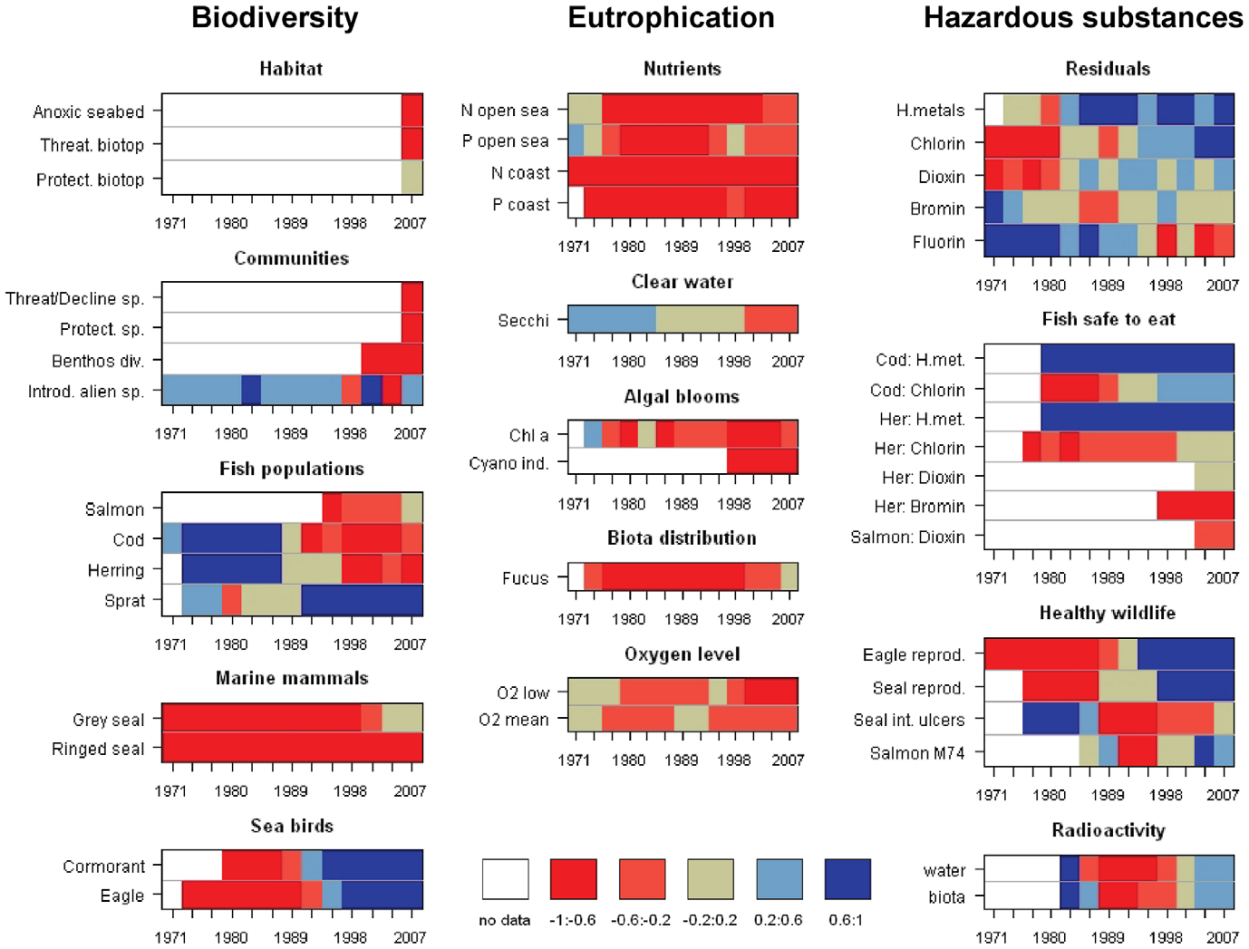
Long-term changes in the state of selected aggregate indicators of the central Baltic Sea. The results are obtained through hierarchical averaging of indicators (see Material and Methods for details) representing the ecological objectives related to biodiversity, eutrophication, and hazardous substances (see Tables S1, S2, S3 and Text S1 for details). The data are averaged by three-year periods and the transformed values (in the scale from −1 to 1) are grouped into five categories shown by colors.

**Figure 4:**
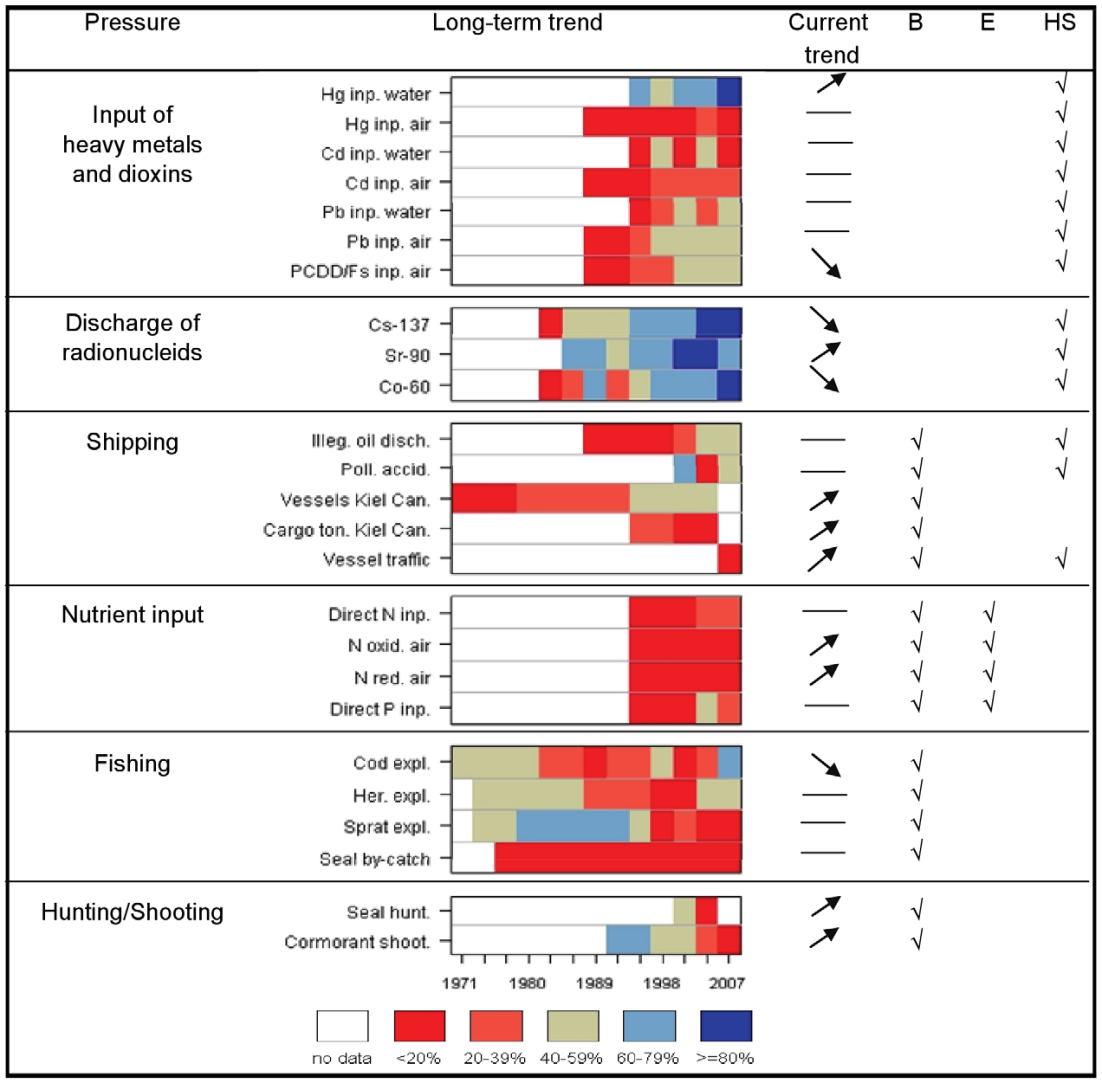
Long-term changes and current trends in indicators representing selected human pressures. Detailed information on indicators and their aggregation is provided in Tables S1, S4 and Text S1). The data showing long-term developments are averaged by three-year periods. The data are presented as a percentage reduction from the highest level observed in the available time series, divided into five categories according to the magnitude of reduction. The current trend is indicated by an arrow showing either an increase or decrease (significant at p<0.1), or no trend (—). The last three columns indicate the direct impact (shown as √) of a given pressure on one or several state indicators of biodiversity (B), eutrophication (E), or hazardous substances (HS).

Long-term performance (since the 1970s) of the state of different components of the ecosystem and environment was presented for the assessment applying hierarchical average for aggregating indicators. Long-term developments supported, in general, the basic conclusions drawn for the current situation. The state of eutrophication has become considerably worse since at least the early 1970s, and only marginal improvement in a few state indicators has been observed in recent years (Figure 3). Despite a substantial reduction in riverine and direct point source inputs of nutrients since the 1990s (Figure 4), the overall status of eutrophication does not indicate a corresponding improvement. In contrast, evaluations of most of the indicators describing the status of hazardous substances have become more positive, despite unfavorable developments in residuals of some brominated and fluorinated compounds in biota (Figure 3). Positive developments are also apparent in several human pressures influencing the status of hazardous substances in the Baltic Sea (Figure 4).

Long-term dynamics of indicators within the biodiversity segment were more variable and changes in the overall biodiversity status were therefore less conclusive. Like the large variability observed in the current status of different biodiversity components (Figure 2), distinct and sometimes opposite dynamics were also apparent (Figure 3). Some indicators displayed a consistently negative status over the decades studied (e.g., ringed seals); populations of several seabirds and also grey seal, which have suffered under heavy human impacts, have recovered with an increase in several times in abundance, but several fish populations exhibited variable and species-specific patterns. Pressures that influence biodiversity were also variable. These pressures include different dynamics and levels of exploitation of fish populations, still high nutrient loads, and increased intensity of maritime transport as well as reduced input of toxic pollution and general progress in nature protection.

### Short-term trends in state and pressures

**Figure 5:**
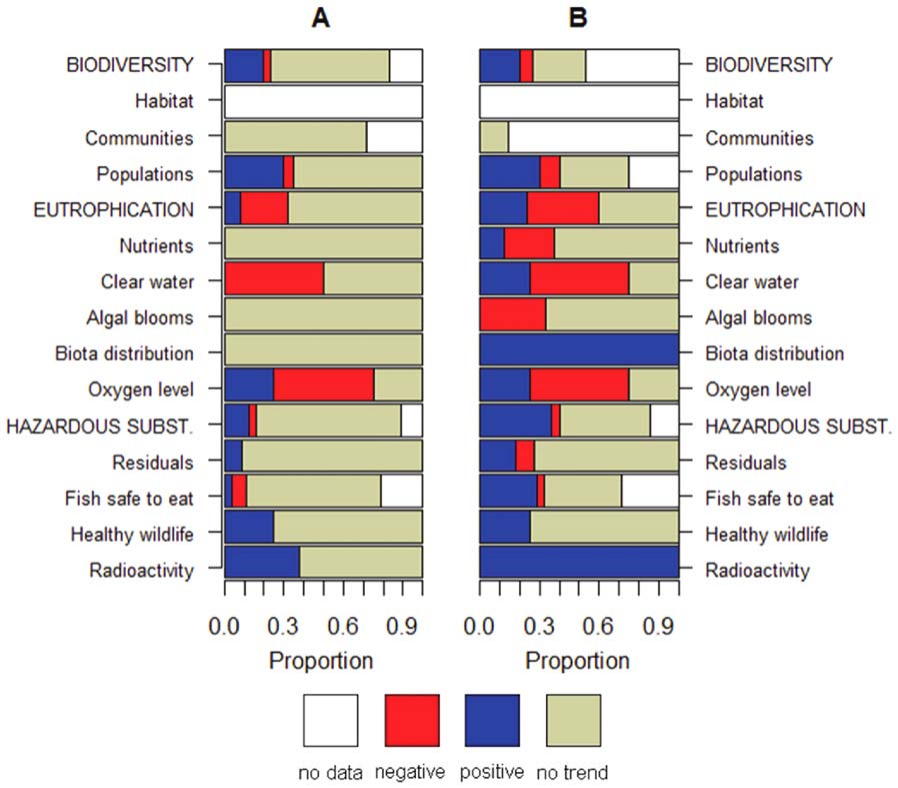
Significant trends in individual state of indicators in the central Baltic Sea. The trends are shown over the last five (panel A) and ten (panel B) years as a proportional distribution between positive, negative, or no trend (shown by colors). No data refers to indicators for which data for less than five or ten years were available.

The analyses of short-term trends (over 5- and 10-year periods) in state indicator time series suggest that among the three strategic goals, eutrophication is of greatest concern. Of the indicators related to eutrophication, a larger proportion (about 35%) exhibits a significant negative trend during the past 10 years, whereas 25% show a significant positive development (Figure 5B). During the recent 5-year period, majority of the indicators related to eutrophication did not show any significant trends; while the few significant trends identified were largely negative (Figure 5A). In contrast, positive trends clearly dominated amongst indicators related to hazardous substances. Similarly to eutrophication, more significant trends in hazardous substances were apparent at a longer (10-year) time scale compared to a 5-year period. Within the biodiversity component, positive trends dominated over negative ones; however, only less than 25% of indicators showed significant trends. This pattern was similar both for the 5- and 10 -year period. However, it should be noted, that several datasets related to biodiversity were short (Figure 3), and trends could therefore not be estimated.

Recent developments in human pressures confirm the worrying signals related to the poor status of eutrophication. Atmospheric inputs have increased in recent years, whereas the inputs of nutrients from point sources have remained relatively unchanged, indicating no significant reduction in nutrient loads in recent times (Figure 4). Recent developments in pressures of human activity related to hazardous substances and biodiversity were less conclusive. The pressures currently increasing in the Baltic Sea include intensified shipping activities and increased inputs of some pollutants (e.g., waterborne input of mercury). In addition, removal of marine organisms at upper trophic levels, such as hunting of grey seals (which was banned for some decades and recently restarted) and shooting of cormorants, is currently increasing, although it is probably still not at a level that affects the status of these populations (Figure 4).

## Discussion

### General

Depending on the spatial/sectoral scales, data availability, and management objectives, several assessment approaches and frameworks related to an ecosystem approach to management of marine environments have been developed in recent years (e.g., [5], [22], [23], [24]). However, most of the related studies focus on the outcomes of evaluations and corresponding management actions, whereas less attention has been paid to the evaluation procedure itself and the methodological challenges associated with it. The process of assessing the environmental status of an ecosystem can be divided roughly into three steps: (i) gathering data and selecting indicators of a sufficiently broad array of components related to given objectives; (ii) defining targets or reference values for indicators; (iii) assessing the overall status by combining information from different indicators. In the following sections we discuss some of the challenges associated with each of these steps, how we have approached these challenges, and which general conclusions could be drawn concerning the importance of these issues for the outcome of an assessment.

### Indicator selection

The central position of indicators at the interface between science and policy points to the importance of their careful selection for management purposes [8]. In practice, identifying appropriate datasets that meet the criteria of an efficient environmental indicator [25] is challenging because of issues such as lack of consistent indicator-evaluation frameworks and institutional commitments for regular data collection [26]. Consequently, a sound management strategy could be to employ a range of indicators to reduce uncertainty resulting from drawing conclusions based on a single indicator [27], [28], [29]. In this paper, we have followed the latter approach, using all available datasets related to the agreed ecological objectives as indicators, given that sufficient knowledge was available to define reference levels.

Our results show that state indicators related to eutrophication and hazardous substances performed relatively homogenously (Figure 2). This suggests that for these segments of the ecosystem that are influenced by a relatively narrow array of drivers and specific kinds of human activities (e.g., eutrophication and hazardous substances), indicator selection and availability are not crucial, as performance of most of the indicators is similar. The situation is different for biodiversity, which is influenced by a variety of human activities both on land and at sea, as well as by climate change, ecological interactions, and conservation measures [30]. Biodiversity status is consequently associated with a broader spectrum of indicators, which may show heterogenous performance as evidenced in the data for the Baltic Sea (Figures 2, 3). For biodiversity, the selection of indicators is therefore crucial because the inclusion or exclusion of certain indicator series might lead to a different evaluation of the overall status.

Adding to the essential complexity of evaluation and management of biodiversity [31], there is a shortage of indicators for some ecological objectives related to biodiversity in the central Baltic Sea (Table S1). There is also a shortage of indicator time series related to human pressures affecting all the three studied segments of environmental status of the central Baltic Sea. This shortage is a general problem also encountered elsewhere (e.g. [32]). Due to greater variability of trends, incomplete coverage of pressures should be considered most problematic for biodiversity and less so for hazardous substances and eutrophication (Figure 4).

### Indicator reference levels and aggregation

In a regulatory context, it is necessary to relate indicators to targets or thresholds that determine the necessity of management actions [7], [10]. However, defining these thresholds and reference states that represent “good environmental status” is one of the greatest challenges to practical implementation of an ecosystem approach to management of marine environments [33]. A “good” status can have many interpretations depending on, for example, public understanding and involvement and different human values [33], [34].

We have tried, where possible, to base our reference levels on scientific criteria and to use the available information from time periods when relevant human pressures were low. Nevertheless, we recognize that several of the thresholds used in this study could also be defined differently. Further, some thresholds might change in future, for example due to climate change, which can potentially result in ecological regime shifts [17], where certain reference levels may become unrealistic to achieve. Uncertainty about reference conditions for management is generally considered one of the greatest weaknesses in existing evaluations of the status of subcomponents of the Baltic ecosystem (e.g., [35]), and future debate in this area should be expected. The reference values we have used in this study could contribute to future work in this area.

An important aspect in reference-based assessment appears to be selection of an indicator aggregation formula. Our analyses showed that the assessment results can be highly sensitive to aggregation rules. The way the indicators are hierarchically arranged influences the assessment results as well, however, these effects were considerably less important than those related to application of different aggregation rules. As shown in our study, application of the widely used “one out – all out” principle (similar to fuzzy AND rule) could easily result in a fully negative overall evaluation for all objectives (Figure 1). The assessment based on this methodology is certainly very conservative from the management perspective and probably ensures a full implementation of precautionary principles. However, a drawback of this approach is that a few strongly negative indicator values could shadow the potentially generally positive state of a given ecological objective. This would make any progress towards improving the environmental status invisible, as long as at least one indicator is showing poor performance. An alternative method that is very often used is application of a simple average across all indicators. The current study evidenced that in situations where a larger number of indicators is available, the choice of applying median or average value in aggregating indicators did not substantially influence the assessment results (Figure 1). However, this might not necessarily be the case when only a few indicators are available, as demonstrated in the example for biodiversity (Figure 1). In such a situation, when applying median of the indicator values, the few indicators showing distinct performance compared to the dominant status, are not taken into account, which in our example resulted in strongly negative overall evaluation of biodiversity status, whereas more positive result was obtained when applying the average of all indicator values (Figure 1).

Simple average (or median) of all indicators is not necessarily the best solution in every circumstance, considering that different indicators meet various screening criteria differently [36]. Individual indicators could be weighted differently in the averaging procedure. However, adequate basis for assigning weights to indicators is usually not available [22], in which case giving all variables equal weight is recommended [37]. Selection of the indicator aggregation formula for a final assessment probably depends on the policy goals and stakeholder preferences. In this study, our intention was to point to the fact that different aggregation rules may give very different evaluation results, and that applying alternative formulas and supplementary methods may be needed to verify the results.

### Trend-based assessment

When sufficient knowledge is lacking to establish quantitative reference levels, and indicator aggregation is posing challenges, a possible alternative approach is trend-based assessment [38]. Under certain conditions, knowledge of the direction of trends in the indicators can be sufficient to support the management decision-making process [39]. In our example, the trend-based assessment results (Figure 5) confirmed conclusions drawn from the reference-based assessments, which applied average or median values in the indicator aggregation process (Figure 1), i.e., poor status of eutrophication, more positive signs for hazardous substances and variable developments within biodiversity. An advantage of a trend-based approach is that it provides a purely observation-based perspective in the performance of indicators, as it is not influenced by potentially subjective or policy-driven definitions of reference values, as well as choices of indicator aggregation methods. Therefore, trend-based analyses would be a good supplement to verify the results of a reference-based assessment.

An important prerequisite, which may often limit conducting trend-based assessments, is the availability of indicator datasets extending for several years back in time. In difference, the current status in relation to indicator thresholds can be evaluated based on data from a few recent years only. However, longer time-series are valuable, also in a refrence-based assessment (Figure 3), for an adequate evaluation of current situation. Further, information on long-term developments could provide an invaluable basis for defining reference conditions (e.g., [40]). Establishing time-series of indicator measurements should therefore be prioritized.

In the analyses investigating short-term trends, a critical aspect to be considered is the length of the time period included in the analysis, which may be important for interpretation of the results. Ecological and environmental datasets are often noisy (e.g., [13]). Thus, indicator trends over a relatively short period of time would seldom be significant. Further, significant developments may be undetectable also on relatively longer time-scales whenindicator values are influenced by ecological processes, which are slow to respond to changes in corresponding pressures (i.e., eutrophication, Figure 5). For example, despite of a large reductions in nutrient inputs to the Baltic Sea since the 1990s (Figure 4), there has been only marginal, if any, measurable improvement in observed nutrient concentrations. Moreover, the status of other eutrophication indicators has generally worsened since then. In contrast, substantially reduced inputs of radionuclides and some toxic compounds and a ban on use of some others (e.g., DDT) have already resulted in significant improvements in the health of structural components of animal populations and communities. Further, abundances of marine animal populations may change rapidly, e.g. the biomass of eastern Baltic cod has more than tripled during recent few years [41]. Thus, indicators influenced by different pressures may respond to changes in these pressures with different time-lags. This is important to take into account for setting a time line for trend-analyses as some recent developments may not appear significant at longer time-scales, whereas gradual changes in some other variables may not be visible at short time scales.

### Conclusions and future challenges

Out of the three BSAP overarching strategic goals, potentially the largest uncertainty is involved in evaluation of the status of biodiversity, mainly because of the variable performance of related indicators. Consequently, evaluation of the status of biodiversity appears to be essentially dependent on the availability and selection of indicator time-series. Therefore, more emphasis should be given in the near future to biodiversity assessments. This work could include analysis of the major trophic levels (i.e., plankton, benthos, fish, birds, and mammals) and different habitats separately, followed by development of formulas for an aggregate biodiversity estimate.

The status of eutrophication of the central Baltic Sea was evaluated to be poor, regardless of indicator selection and assessment methodology. The status of hazardous substances appears to be the best among the three strategic goals defined by BSAP. These conclusions are generally in line with the HELCOM initial holistic assessment [14]. Though, it should be noted that a strongly negative status of hazardous substances could be obtained, when applying most conservative indicator aggregation rules. Concerning all segments of environmental status, the assessment results are sensitive to reference level settings and to indicator aggregation rules. Trend-based assessment is therefore recommended as a useful supplement to reference-based evaluation.

Much of the indicator development so far has concentrated on the ecosystem state, while establishing links between state and pressure largely remains a future challenge (e.g., [14], [32], [42]). There is a general need to improve our basic understanding of links between changes in external human drivers and the structure and functioning of ecosystems. This would, amongst others, allow setting realistic deadlines, when an improvement in the environmental status may be expected, after a particular pressure has been reduced. Such research should be given priority in further development of indicator-based assessment and management of the Baltic Sea. In addition, in those sectors where unacceptable situations or undesired developments continue to occur, establishing new and more ambitious management targets might be needed.

Most advances in the work of developing indicators for an ecosystem approach to management of the marine environment have been related to ecological indicators, and less information is available for socioeconomic and governance aspects. Increasing demand for indicators in the two latter categories [43], [44] also calls for future emphasis on these categories for the Baltic Sea. The available tools, such as the approach we have used in this study, would allow for coherent integration of the entire spectrum of indicators related to an ecosystem approach to management of marine environments [22], which would then allow for a holistic evaluation of the progress in implementing the EAM in the Baltic Sea.

## Supporting Information

Table S1 Description of indicators. Description of state and pressure indicators with their acronyms as used in the paper, the time period for which the indicator data were included in the paper and data sources. For cited references, see Text S1.
(DOC)

Table S2 Indicator thresholds. Threshold values corresponding to reference, acceptable or bad status for each state indicator (shown by their acronyms; see Table S1 for description of indicators), and rationale for the defined thresholds. For cited references see Text S1.
(DOC)

Table S3 Structure for aggregating state indicators. Hierarchical structure for aggregating state indicators (shown by their acronyms; see Table S1 for indicator descriptions) into Objectives and Goals via up to three intermediate steps (Steps 1–3).
(DOC)

Table S4 Structure for aggregating pressure indicators. Hierarchical structure for aggregating pressure indicators (shown by their acronyms; see Table S1 for indicator descriptions) by sources of pressure via an intermediate step (Step 1), where relevant.
(DOC)

Text S1 List of references cited in Tables S1, S2, S3, S4.
(DOC)
