# Supplementary material for: Methodological Challenges in Assessing the Environmental Status of a Marine Ecosystem: Case Study of the Baltic Sea
Source: PLoS One. 2011 Apr 29;6(4):e19231. doi: 10.1371/journal.pone.0019231 (PMC3084783; doi:10.1371/journal.pone.0019231)
Supplement: Table S1 — Description of indicators. Description of state and pressure indicators with their acronyms as used in the paper, the time period for which the indicator data were included in the paper and data sources. For cited references, see Text S1. (DOC) [file pone.0019231.s001.doc]

| **Acronym** | **Type** | **Indicator description** | **Period** | **Source** |
| --- | --- | --- | --- | --- |
| Hg Guill. | State | Concentration of Hg in guillemot eggs (ng/g fresh w.) in St. Karlsö | 1975-2006 | [1] |
| sDDT Guill. | State | Combined concentration of DDTs in guillemot eggs (μg/g) in St. Karlsö | 1971-2007 | [2] |
| DDE Eagle | State | Concentration of DDE in white tailed sea eagle eggs (μg/g lipid w.) | 1970-2005 | [3] |
| sPCB Guill. | State | Combined concentration of PCBs in guillemot eggs (μg/g lipid w.) in St. Karlsö | 1971-2006 | [1] |
| PCB Eagle | State | Concentration of total PCB in white tailed sea eagle eggs (μg/g lipid w.) | 1970-2005 | [3] |
| b_HCH Guill. | State | Concentration of b-HCH in guillemot eggs (μg/g lipid w.) in St. Karlsö | 1988-2007 | [2] |
| HCB Guill. | State | Concentration of HCB in guillemot eggs (μg/g lipid w.) in St. Karlsö | 1979-2007 | [2] |
| TCDD Guill. | State | Concentration of TCDD-equivalents in guillemot eggs (pg/g fat) in St. Karlsö | 1971-2007 | [2] |
| BDE_47 Guill. | State | Concentration of BDE-47 in guillemot eggs (ng/g lipid w.) in St. Karlsö | 1971-2007 | [1] |
| HBCD Guill. | State | Concentration of HBCD in guillemot eggs (ng/g lipid w.) in St Karlsö | 1971-2007 | [1] |
| PFOS Guill. | State | Concentration of PFOS in guillemot eggs (μg/g) in St. Karlsö | 1971-2007 | [2] |
| Cd Cod | State | Concentration of Cd in cod liver (μg/g dry w.) in Gotland | 1981-2006 | [2] |
| Cu Cod | State | Concentration of Cu in cod liver (μg/g dry w.) in Gotland | 1981-2006 | [1] |
| Hg Cod | State | Concentration of Hg in cod muscle (ng/g fresh w.) in Gotland | 1979-2006 | [1] |
| Pb Cod | State | Concentration of Pb in cod liver (μg/g dry w.) in Gotland | 1981-2005 | [1] |
| Zn Cod | State | Concentration of Zn in cod liver (μg/g dry w.) in Gotland | 1981-2006 | [1] |
| sDDT cod | State | Concentration of DDE in cod liver (μg/g lipid w.) in Gotland | 1980-2005 | [1] |
| Cd Her_Ut | State | Concentration of Cd in herring liver (μg/g dry w.) in Utlangan | 1981-2006 | [1] |
| Cd Her_Ls | State | Concentration of Cd in herring liver (μg/g dry w.) in Landsort | 1981-2006 | [1] |
| Cu Her_Ut | State | Concentration of Cu in herring liver (μg/g dry w.) in Utlangan | 1981-2006 | [1] |
| Cu Her_Ls | State | Concentration of Cu in herring liver (μg/g dry w.) in Landsort | 1981-2006 | [1] |
| Hg Her_Ut | State | Concentration of Hg in herring muscle (ng/g fresh w.) in Utlangan | 1980-2006 | [1] |
| Hg Her_Ls | State | Concentration of Hg in herring muscle (ng/g fresh w.) in Landsort | 1981-2006 | [1] |
| Pb Her_Ut | State | Concentration of Pb in herring liver (μg/g dry w.) in Utlangan | 1981-2006 | [1] |
| Pb Her_Ls | State | Concentration of Pb in herring liver (μg/g dry w.) in Landsort | 1981-2006 | [1] |
| Zn Her_Ut | State | Concentration of Zn in herring liver (μg/g dry w.) in Utlangan | 1981-2006 | [1] |
| Zn Her_Ls | State | Concentration of Zn in herring liver (μg/g dry w.) in Landsort | 1981-2006 | [1] |
| sDDT Her_Ut | State | Combined concentration of DDTs in herring muscle (μg/g lipid w.) in Utlangan | 1979-2005 | [1] |
| sDDT Her_Ls | State | Combined concentration of DDTs in herring muscle (μg/g lipid w.) in Landsort | 1980-2005 | [1] |
| sPCB Her_Ut | State | Combined concentration of PCBs in herring muscle (μg/g lipid w.) in Utlangan | 1980-2007 | [2] |
| sPCB Her_Ls | State | Combined concentration of PCBs in herring muscle (μg/g lipid w.) in Landsort | 1978-2007 | [2] |
| TCDD Her_SB | State | Dioxin ( WHOPCDD/F-TEQ) content in herring muscle (ng/kg fresh w.) in south of Bornholm | early 2000s | [4] |
| TCDD Her_EB | State | Dioxin ( WHOPCDD/F-TEQ) content in herring muscle (ng/kg fresh w.) in east of Bornholm | early 2000s | [4] |
| TCDD Her_POL | State | Dioxin ( WHOPCDD/F-TEQ) content in herring muscle (ng/kg fresh w.) at Polish coast | early 2000s | [4] |
| TCDD Her_LAT | State | Dioxin ( WHOPCDD/F-TEQ) content in herring muscle (ng/kg fresh w.) at Latvian coast | early 2000s | [4] |
| TCDD Her_SG | State | Dioxin ( WHOPCDD/F-TEQ) content in herring muscle (ng/kg fresh w.) in south of Gotland | early 2000s | [4] |
| HBCD her_Ut | State | Concentration of HBCD in herring muscle (ng/g lipid w.) in Utlangan | 1999-2007 | [2] |
| HBCD her_Ls | State | Concentration of HBCD in herring muscle (ng/g lipid w.) in Landsort | 1999-2007 | [2] |
| TCDD salm. | State | Typical dioxin levels in Baltic wild salmon (ng WHO-TEQ/kg fresh weight) | early 2000s | [4] |
| Eagle repr. suc_MWP | State | Breeding success (%) of white-tailed sea eagle in Mecklenburg-Western Pomerania, Germany | 1973-2008 | [3] |
| Eagle repr. suc_BP | State | Breeding success (%) of white-tailed sea eagle at Baltic Proper west coast | 1970-2007 | [3] |
| Eagle br. size_BP | State | Mean nestling brood size of white-tailed sea eagle at Baltic Proper west coast | 1970-2008 | [3] |
| Seal ut. obstr. | State | Prevalence of uterine obstructions in female grey seals >4 years | 1977-2008 | [5] |
| Seal ut. leiom. | State | Prevalence of uterine leiomyomas in female grey seals >4 years | 1977-2008 | [5] |
| Seal pct pregn. | State | Prevalence of pregnant 4-20 years old grey seals | 1977-2008 | [5] |
| Salmon M74 | State | M74 frequency (%) as a proportion of M74 females (partial or total offspring M74 mortality) belonging to reared populations of Baltic salmon | 1985-2008 | [6] |
| Seal int. ulcers | State | Prevalence of intestinal ulcers in juvenile (age 1-3) grey seals | 1977-2008 | [5] |
| Cs_137_BPN | State | Concentration of Cs-137 in surface water (Bq/m^3^) in northern Baltic Proper | 1984-2006 | [7] |
| Cs_137_BPS | State | Concentration of Cs-137 in surface water (Bq/m^3^) in southern Baltic Proper | 1984-2006 | [7] |
| Cs_137_BPM | State | Concentration of Cs-137 in surface water (Bq/m^3^) in mid Baltic Proper | 1984-2006 | [7] |
| Cs_137 Her_BPN | State | Concentration of Cs-137 in herring muscle (Bq/kg wet w.) in northern Baltic Proper | 1985-2002 | [7] |
| Cs_137 Her_BPS | State | Concentration of Cs-137 in herring muscle (Bq/kg wet w.) in southern Baltic Proper | 1984-2006 | [7] |
| Cs_137 Flatf_EB | State | Concentration of Cs-137 in flatfish muscle (Bq/kg wet w.) in eastern Baltic | 1985-2006 | [7] |
| Sr_90 pike_EB | State | Concentration of Sr-90 in pike muscle (Bq/kg wet w.) in eastern Baltic | 1984-2006 | [7] |
| Cs_137 Fuc_WB | State | Concentration of Cs-137 in bladder wrack (Bq/kg wet w.) in western Baltic | 1985-2006 | [7] |
| DIN open | State | Winter mean (Dec-March) disscolved inorganic nitrogen in surface water (0-10m) in open Baltic Proper (μmol l^-1)^ | 1970-2006 | [8] |
| TN open | State | Annual mean of total nitrogen in surface water (0-10m) in open Baltic Proper (μmol l^-1^) | 1970-2006 | [8] |
| DIP open | State | Winter mean (Dec-March) disscolved inorganic phosphorus in surface water (0-10m) in open Baltic Proper (μmol l^-1)^ | 1970-2006 | [8] |
| TP open | State | Annual mean of total phosphorus in surface water (0-10m) in open Baltic Proper (μmol l^-1^) | 1970-2006 | [8] |
| DIN coast | State | Winter mean (Dec-March) disscolved inorganic nitrogen in surface water (0-10m) in coastal Baltic Proper (μmol l^-1)^ | 1972-2006 | [8] |
| TN coast | State | Annual mean of total nitrogen in surface water (0-10m) in coastal Baltic Proper (μmol l^-1^) | 1974-2006 | [8] |
| DIP coast | State | Winter mean (Dec-March) disscolved inorganic phosphorus in surface water (0-10m) in coastal Baltic Proper (μmol l^-1)^ | 1974-2006 | [8] |
| TP coast | State | Annual mean of total phosphorus in surface water (0-10m) in coastal Baltic Proper (μmol l^-1^) | 1975-2006 | [8] |
| Secchi_EG | State | Water transparency in June-September measured as Secchi depth (m) fitted with non-linear smoothing in Eastern Gotland Basin | 1970-2006 | [9] |
| Secchi_WG | State | Water transparency in June-September measured as Secchi depth (m) fitted with non-linear smoothing in Western Gotland Basin | 1970-2006 | [9] |
| Secchi_NBP | State | Water transparency in June-September measured as Secchi depth (m) fitted with non-linear smoothing in Northern Baltic Proper | 1970-2006 | [9] |
| Secchi_BB | State | Water transparency in June-September measured as Secchi depth (m) fitted with non-linear smoothing in Bornholm Sea | 1970-2006 | [9] |
| Chl a open | State | Annual mean (January-December) of Chl a concentration in open Baltic Proper | 1974-2006 | J. Carstensen, unpublished |
| Chl a coast | State | Annual mean (January-December) of Chl a concentration in coastal Baltic Proper | 1976-2006 | J. Carstensen, unpublished |
| Cyano ind. | State | Cyanobacterial bloom abundance-based index (Aphanizomenon flos-aquae, Nodularia spumigena) in the Travemünde-Helsinki transect | 1997-2007 | [10] |
| Fucus mean | State | Average depth limit (m) of the distribution of *Fucus vesiculosus* in Baltic Proper | 1974-2007 | [8] |
| Fucus max | State | Observed absolute maximum depth (m) of distribution of *Fucus vesiculosus* in Baltic Proper | 1974-2007 | [8] |
| O2 low_EG | State | Autumn bottom oxygen concentration (ml l^-1^) in Eastern Gotland Basin as a 5-year running mean of the lowest 25% of the data | 1970-2006 | [8] |
| O2 low_WG | State | Autumn bottom oxygen concentration (ml l^-1^) in Western Gotland Basin as a 5-year running mean of the lowest 25% of the data | 1970-2006 | [8] |
| O2 low_NBP | State | Autumn bottom oxygen concentration (ml l^-1^) in Northern Baltic Proper as a 5-year running mean of the lowest 25% of the data | 1970-2006 | [8] |
| O2 low_SBP | State | Autumn bottom oxygen concentration (ml l^-1^) in Southern Baltic Proper as a 5-year running mean of the lowest 25% of the data | 1970-2006 | [8] |
| O2 mean_EG | State | Autumn bottom oxygen concentration (ml l^-1^) in Eastern Gotland Basin as a 5-year running mean | 1970-2006 | [8] |
| O2 mean_WG | State | Autumn bottom oxygen concentration (ml l^-1^) in Western Gotland Basin as a 5-year running mean | 1970-2006 | [8] |
| O2 mean_NBP | State | Autumn bottom oxygen concentration (ml l^-1^) in Northern Baltic Proper as a 5-year running mean | 1970-2006 | [8] |
| O2 mean_SBP | State | Autumn bottom oxygen concentration (ml l^-1^) in Southern Baltic Proper as a 5-year running mean | 1970-2006 | [8] |
| Anoxic seabed | State | Anoxic seabed area (10000 km3) | mid-2000s | [11] |
| Threat. biotop | State | Number of threatened biotopes in central Baltic | mid-2000s | [12] |
| Protect. biotop | State | Number of threatened biotopes in central Baltic which are protected by MPAs | 2010 | [13] |
| Threat/Decline sp. | State | Number of threatened and declining species in total Baltic Sea | mid-2000s | [12] |
| Protect. sp. | State | Number of threatened species which are protected in total Baltic Sea | 2010 | [13] |
| Benthos div_BB | State | Average number of benthic invertebrate species in Bornholm Basin | 2000-2006 | [11] |
| Benthos div_SG | State | Average number of benthic invertebrate species in SE Gotland Basin | 2000-2006 | [11] |
| Benthos div_NG | State | Average number of benthic invertebrate species in NE Gotland Basin | 2000-2006 | [11] |
| Benthos div_NBP | State | Average number of benthic invertebrate species in N Baltic Proper | 2000-2006 | [11] |
| Introd. alien sp. | State | Introduction of new species established in the Baltic Sea | 1970-2008 | [14] |
| Smolt prod_Moerr | State | Smolt production in Mörrumsån river | 1996-2008 | [6] |
| Smolt prod_Emån | State | Smolt production in Emån river | 1996-2008 | [6] |
| Smolt prod_Irbe | State | Smolt production in Irbe river | 2001-2008 | [6] |
| Smolt prod_Venta | State | Smolt production in Venta river | 2001-2008 | [6] |
| Smolt prod_Saka | State | Smolt production in Saka river | 2001-2008 | [6] |
| Smolt prod_Uzava | State | Smolt production in Uzava river | 2001-2008 | [6] |
| Smolt prod_Barta | State | Smolt production in Barta river | 2001-2008 | [6] |
| Smolt prod_Nemunas | State | Smolt production in Nemunas river | 1996-2008 | [6] |
| Cod ssb | State | Spawning stock biomass of eastern Baltic cod (kt) | 1970-2008 | [15] |
| Cod rec | State | Recruitment (numbers at age 2; mill.) of eastern Baltic cod | 1970-2008 | [15] |
| Her. ssb | State | Spawning stock biomass of herring in SD 25-29 (kt; excl. Gulf of Riga) | 1974-2008 | [15] |
| Her. rec | State | Recruitment (age 1; mill.) of herring in SD 25-29 (excl. Gulf of Riga) | 1974-2008 | [15] |
| Spr. ssb | State | Spawning stock biomass of sprat (kt;SD 22-32) | 1974-2008 | [15] |
| Spr. rec | State | Recruitment (age 1; mill.) of sprat (SD 22-32) | 1974-2008 | [15] |
| Grey seal | State | Population abundance (numbers) of grey seal | 1970-2008 | [16,17,18] |
| Ringed seal | State | Population abundance (numbers) of ringed seal | 1970-2008 | [11,16] |
| cormorant_DK | State | Great cormorant population (number of breeding pairs) in Denmark (Southern Baltic Sea) | 1980-2008 | [19] |
| cormorant_MWP | State | Great cormorant population (number of breeding pairs) in Mecklenburg-Western Pomerania (southern Baltic Sea) | 1980-2008 | [19] |
| cormorant_SH | State | Great cormorant population (number of breeding pairs) in Schleswig-Holstein (southern Baltic Sea) | 1980-2008 | [19] |
| Eagle | State | Number of territorial pairs of white-tailed eagle (*Haliaeetus albicilla*) in Mecklenburg-Western Pomerania | 1973-2008 | [20] |
| Hg inp. air | Pressure | Atmospheric deposition of Hg (tonnes/year) to Baltic Proper | 1990-2007 | [21] |
| Cd inp. air | Pressure | Atmospheric deposition of Cd (tonnes/year) to Baltic Proper | 1990-2007 | [21] |
| Pb inp. air | Pressure | Atmospheric deposition of Pb (tonnes/year) to Baltic Proper | 1990-2007 | [21] |
| Hg inp. water | Pressure | Waterborne inputs of Hg (tonnes/year) to the Baltic Sea | 1994-2006 | [22] |
| Cd inp. water | Pressure | Waterborne inputs of Cd (tonnes/year) to the Baltic Sea | 1994-2006 | [22] |
| Pb inp. water | Pressure | Waterborne inputs of Pb (tonnes/year) to the Baltic Sea | 1994-2006 | [22] |
| PCDD/Fs inp. air | Pressure | Atmospheric deposition of PCDD/Fs (g TEQ/year) to Baltic Proper | 1990-2007 | [23] |
| Cs_137_Oskh | Pressure | Annual liquid discharge of caesium-137 (Bq) from Oskarshamn nuclear power plant | 1984-2007 | [24] |
| Cs_137_Studs | Pressure | Annual liquid discharge of caesium-137 (Bq) from Studsvik nuclear power plant | 1984-2007 | [24] |
| Sr_90_Oskh | Pressure | Annual liquid discharge of strontium-90 (Bq) from Oskarshamn nuclear power plant | 1985-2007 | [24] |
| Sr_90_Studs | Pressure | Annual liquid discharge of strontium-90 (Bq) from Studsvik nuclear power plant | 1985-2007 | [24] |
| Co_60_Oskh | Pressure | Annual liquid discharge of cobalt-60 (Bq) from Oskarshamn nuclear power plant | 1984-2007 | [24] |
| Co_60_Studs | Pressure | Annual liquid discharge of cobalt-60 (Bq) from Studsvik nuclear power plant | 1984-2007 | [24] |
| Direct N inp. | Pressure | Riverine and direct point source total nitrogen inputs (t) into the Baltic Proper | 1994-2006 | [8] |
| N oxid. air | Pressure | Atmospheric deposition of oxidized nitrogen (kt/year) to the Baltic Proper | 1995-2007 | [25] |
| N red. air | Pressure | Atmospheric deposition of reduced nitrogen (kt/year) to the Baltic Proper | 1995-2007 | [25] |
| Direct P inp. | Pressure | Riverine and direct point source total prosphorus inputs (t) into the Baltic Proper | 1994-2006 | [8] |
| Illeg. oil disch. | Pressure | Number of detected oil spills during aerial surveillance | 1988-2007 | [26] |
| Poll. accid. | Pressure | Number of reported ship accidents resulting in oil pollution | 2000-2008 | [27] |
| Vessels Kiel Can. | Pressure | Number of all non-pleasure boats passing through the Kiel Canal | 1970-2004 | [28] |
| Cargo ton. Kiel Can. | Pressure | Total cargo tonnage of ships passing throught the Kiel Canal | 1996-2003 | [28] |
| Vessel traffic | Pressure | Number of ships crossing fixed AIS (Automatic Identification System) lines in the Baltic Sea | 2006-2008 | [29] |
| Cod expl. ad. | Pressure | Fishing mortality on adult cod (ages 4-7) | 1970-2008 | [15] |
| Cod expl. rec. | Pressure | Fishing mortality on cod recruitment (age 2) | 1970-2008 | [15] |
| Her. expl. | Pressure | Fishing mortality on herring (ages 3-6) | 1970-2008 | [15] |
| Spr. expl. | Pressure | Fishing mortality on sprat (ages 3-5) | 1970-2008 | [15] |
| Seal by-catch | Pressure | Grey seals killed by fishing gear in Sweden and Finland as percentage of total seal mortality (excluding hunting) | 1977-2008 | [5] |
| Seal hunt. | Pressure | Number of grey seals hunted | 2000-2005 | [5] |
| Corm. shoot. DK | Pressure | Shooting of cormorants in Denmark (in numbers) | 1993-2008 | [19] |
| Corm. shoot. MWP | Pressure | Shooting of cormorants in Mecklenburg-Western Pomerania, southern Baltic (in numbers) | 1993-2008 | [19] |
| Corm. shoot. SH | Pressure | Shooting of cormorants in Schleswig-Holstein, southern Baltic (in numbers) | 1993-2008 | [19] |
| Corm. shoot. EST | Pressure | Shooting of cormorants in Estonia (in numbers) | 1993-2008 | [19] |
