# Supplementary material for: Methodological Challenges in Assessing the Environmental Status of a Marine Ecosystem: Case Study of the Baltic Sea
Source: PLoS One. 2011 Apr 29;6(4):e19231. doi: 10.1371/journal.pone.0019231 (PMC3084783; doi:10.1371/journal.pone.0019231)
Supplement: Table S2 — Indicator thresholds. Threshold values corresponding to reference, acceptable or bad status for each state indicator (shown by their acronyms; see Table S1 for description of indicators), and rationale for the defined thresholds. For cited references see Text S1. (DOC) [file pone.0019231.s002.doc]

| **Indicator** | **Reference** | **Acceptable** | **Bad** | **Rationale** |
| --- | --- | --- | --- | --- |
| Hg Guill. | 230 | 500 |  | R: min. level observed in available data (since 1970s); A: max. level allowed in selected food in EU [30] |
| sDDT Guill. | 0 |  | 300 | R: does not occur in nature; B: observed average level before mid-1980s, after which higher trophic levels started to recover [31] |
| DDE Eagle | 0 |  | 500 | R: does not occur in nature; B: observed average level before mid-1980s, when eagle productivity was low due to pollution [3, 20] |
| sPCB Guill. | 0 |  | 200 | R: does not occur in nature; B: observed average level before mid-1980s, after which higher trophic levels started to recover [31] |
| PCB Eagle | 0 |  | 900 | R: does not occur in nature; B: observed average level before mid-1980s, when eagle productivity was low due to pollution [3,20] |
| B_HCH Guill. | 0 |  | 1.2 | R: does not occur in nature; B: max. level observed in available data (in late 1980s) |
| HCB Guill. | 0 |  | 4.5 | R: does not occur in nature; B: max. level observed in available data (in late 1970s) |
| TCDD Guill. | 0 |  | 3000 | R: does not occur in nature; B: max. level observed in available data (late 1960s) |
| BDE_47 Guill. | 0 |  | 1000 | R: does not occur in nature; B: max. level observed in available data (mid-1980s) |
| HBCD Guill. | 0 |  | 200 | R: does not occur in nature; B: max. level observed in available data (mid-2000s) |
| PFOS Guill. | 0 |  | 1300 | R: does not occur in nature; B: max. level observed in available data (mid 2000s) |
| Cd Cod | 0.02 | 6 |  | R: min. level observed since 1980s; A: The highest concentration (level 4) allowed for human consumption of fish in Sweden (A. Bignert, unpublished) |
| Cu Cod | 10 | 80 |  | R: min. level observed since 1980s; A: The highest concentration (level 4) allowed for human consumption of fish in Sweden (A. Bignert, unpublished) |
| Hg Cod | 15 | 500 |  | R: min. level observed since 1980s; A: max. level allowed in selected food in EU [30] |
| Pb Cod | 0.02 | 0.8 |  | R: min. level observed since 1980s; A: The highest concentration (level 4) allowed for human consumption of fish in Sweden (A. Bignert, unpublished) |
| Zn Cod | 21 | 500 |  | R: min. level observed since 1980s; A: The highest concentration (level 4) allowed for human consumption of fish in Sweden (A. Bignert, unpublished) |
| sDDT cod | 0 | 1 |  | R: does not occur in nature; A: max. level allowed for meat products for human consumption in EU [32] |
| Cd Her_Ut | 0.7 | 6 |  | R: min. level observed since 1980s; A: The highest concentration (level 4) allowed for human consumption of fish in Sweden (A. Bignert, unpublished) |
| Cd Her_Ls | 0.7 | 6 |  | R: min. level observed since 1980s; A: The highest concentration (level 4) allowed for human consumption of fish in Sweden (A. Bignert, unpublished) |
| Cu Her_Ut | 7 | 80 |  | R: min. level observed since 1980s; A: The highest concentration (level 4) allowed for human consumption of fish in Sweden (A. Bignert, unpublished) |
| Cu Her_Ls | 7 | 80 |  | R: min. level observed since 1980s; A: The highest concentration (level 4) allowed for human consumption of fish in Sweden (A. Bignert, unpublished) |
| Hg Her_Ut | 9 | 500 |  | R: min. level observed since 1980s; A: max. level allowed in selected food in EU [30] |
| Hg Her_Ls | 9 | 500 |  | R: min. level observed since 1980s; A: max. level allowed in selected food in EU [31] |
| Pb Her_Ut | 0.07 | 0.8 |  | R: min. level observed since 1980s; A: The highest concentration (level 4) allowed for human consumption of fish in Sweden (A. Bignert, unpublished) |
| Pb Her_Ls | 0.07 | 0.8 |  | R: min. level observed since 1980s; A: The highest concentration (level 4) allowed for human consumption of fish in Sweden (A. Bignert, unpublished) |
| Zn Her_Ut | 70 | 500 |  | R: min. level observed since 1980s; A: The highest concentration (level 4) allowed for human consumption of fish in Sweden (A. Bignert, unpublished) |
| Zn Her_Ls | 70 | 500 |  | R: min. level observed since 1980s; A: The highest concentration (level 4) allowed for human consumption of fish in Sweden (A. Bignert, unpublished) |
| sDDT Her_Ut | 0 | 1 |  | R: does not occur in nature; A: max. level allowed for meat products for human consumption in [32] |
| sDDT Her_Ls | 0 | 1 |  | R: does not occur in nature; A: max. level allowed for meat products for human consumption in EU [32] |
| sPCB Her_Ut | 0 | 0.2 |  | R: does not occur in nature; A: max. level allowed in selected food in EU [33] |
| sPCB Her_Ls | 0 | 0.2 |  | R: does not occur in nature; A: max. level allowed in selected food in EU [33] |
| TCDD Her_SB | 0 | 4 |  | R: does not occur in nature; A: max. level allowed in selected food in EU [34] |
| TCDD Her_EB | 0 | 4 |  | R: does not occur in nature; A: max. level allowed in selected food in EU [34] |
| TCDD Her_POL | 0 | 4 |  | R: does not occur in nature; A: max. level allowed in selected food in EU [34] |
| TCDD Her_LAT | 0 | 4 |  | R: does not occur in nature; A: max. level allowed in selected food in EU [34] |
| TCDD Her_SG | 0 | 4 |  | R: does not occur in nature; A: max. level allowed in selected food in EU [34] |
| HBCD her_Ut | 0 | 4 |  | R: does not occur in nature; A: max. level allowed in selected food in EU [34] |
| HBCD her_Ls | 0 | 4 |  | R: does not occur in nature; A: The highest concentration (level 4) allowed for human consumption of fish in Sweden (A. Bignert, unpublished) |
| TCDD salm. | 0 | 4 |  | R: does not occur in nature; A: The highest concentration (level 4) allowed for human consumption of fish in Sweden (A. Bignert, unpublished) |
| Eagle repr. suc_MWP | 61 |  | 44 | R: lower level of confidence limits for normal reproduction (B. Helander, unpublished); B: 25% reduction from normal reproduction (B. Helander, unpublished) |
| Eagle repr. suc_BP | 61 |  | 44 | R: lower level of confidence limits for normal reproduction (B. Helander, unpublished); B: 25% reduction from normal reproduction (B. Helander, unpublished) |
| Eagle br. size_BP | 1.61 |  | 1.24 | R: lower level of confidence limits for normal reproduction (B. Helander, unpublished); B: 25% reduction from normal reproduction (B. Helander, unpublished) |
| Seal ut. obstr. | 0 |  | 40 | R: recent observed level when population has been recovering; B: observed level in the 1970s-1980s when pollution was the highest [5] |
| Seal ut. leiom. | 2 |  | 45 | R: recent observed level when population has been recovering; B: observed level in the 1970s-1980s when pollution was the highest [5] |
| Seal pct pregn. | 77 |  | 9 | R: recent observed level when population has been recovering; B: observed level in the 1970s-1980s when pollution was the highest [5] |
| Salmon M74 | 3 |  | 50 | R: observed min. level; B: observed max. level (1992-1996) when salmon was seriously affected [6] |
| Seal int. ulcers | 15 |  | 55 | R: observed min. level (late 1970s); B: observed max. level (late 1980s-early 1990s) [5] |
| Cs_137_BPN | 15 |  | 100 | R: level prior to Chernobyl accident [7]; B: average observed level after Chernobyl accident [7] |
| Cs_137_BPS | 15 |  | 100 | R: level prior to Chernobyl accident [7]; B: average observed level after Chernobyl accident [7] |
| Cs_137_BPM | 15 |  | 100 | R: level prior to Chernobyl accident [7]; B: average observed level after Chernobyl accident [7] |
| Cs_137 Her_BPN | 2.5 |  | 20 | R: level prior to Chernobyl accident [7]; B: average observed level after Chernobyl accident [7] |
| Cs_137 Her_BPS | 2.5 |  | 12 | R: level prior to Chernobyl accident [7]; B: average observed level after Chernobyl accident [7] |
| Cs_137 Flatf_EB | 2.5 |  | 12 | R: level prior to Chernobyl accident [7]; B: average observed level after Chernobyl accident [7] |
| Sr_90 pike_EB | 5 |  | 30 | R: level prior to Chernobyl accident [7]; B: average observed level after Chernobyl accident [7] |
| Cs_137 Fuc_WB | 10 |  | 50 | R: level prior to Chernobyl accident [7]; B: average observed level after Chernobyl accident [7] |
| DIN open | 2 | 3.0 |  | R: observed lowest level in the northern Baltic [35]; A: 50% deviance from reference |
| TN open | 9.5 | 14.3 |  | R: average level in Danish Straits and the Sound [35]; A: 50% deviance from reference |
| DIP open | 0.25 | 0.4 |  | R: identified reference conditions in Gotland Basin [35]; A: 50% deviance from reference |
| TP open | 0.37 | 0.6 |  | R: average level in Danish Straits and the Sound [35]; A: 50% deviance from reference |
| DIN coast | 2 | 3.0 |  | R: observed lowest level in the northern Baltic [35]; A: 50% deviance from reference |
| TN coast | 9.5 | 14.3 |  | R: average level in Danish Straits and the Sound [35]; A: 50% deviance from reference |
| DIP coast | 0.25 | 0.4 |  | R: identified reference conditions in Gotland Basin [35]; A: 50% deviance from reference |
| TP coast | 0.37 | 0.6 |  | R: average level in Danish Straits and the Sound [35]; A: 50% deviance from reference |
| Secchi_EG | 8 | 6 |  | R: pre-industrial level (early 1900s); A: target defined by HELCOM [36] |
| Secchi_WG | 8 | 6 |  | R: pre-industrial level (early 1900s); A: target defined by HELCOM [36] |
| Secchi_NBP | 9 | 7 |  | R: pre-industrial level (early 1900s); A: target defined by HELCOM [36] |
| Secchi_BB | 9 | 7 |  | R: pre-industrial level (early 1900s); A: target defined by HELCOM [36] |
| Chl a open | 1 | 1.5 |  | R: observed min. level in Bothinan Bay (J. Carstensen, unpublished) indicating low trophic status; A: 50% deviation from reference |
| Chl a coast | 1 | 1.5 |  | R: observed min. level in Bothinan Bay (J. Carstensen, unpublished) indicating low trophic status; A: 50% deviation from reference |
| Cyano ind. | 15 | 22.5 |  | R: calculated appr. index value for natural levels in 19th century based on [37]; A: 50% deviation from reference |
| Fucus mean | 9 | 6.8 |  | R: Mid-1940s level [38]; A: 25% deviation from reference |
| Fucus max | 11.5 | 8.6 |  | R: Mid-1940s level [38]; A: 25% deviation from reference |
| O2 low_EG | 3.5 | 0 |  | R: defined by [8]; A: no hydrogen sulfide formation |
| O2 low_WG | 3.5 | 0 |  | R: defined by [8]; A: no hydrogen sulfide formation |
| O2 low_NBP | 3.5 | 0 |  | R: defined by [8]; A: no hydrogen sulfide formation |
| O2 low_SBP | 3.5 | 0 |  | R: defined by [8]; A: no hydrogen sulfide formation |
| O2 mean_EG | 3.5 | 2.1 |  | R:defined by [8]; A: above the level corresponding to hypoxia of 2 ml l^-1^ [39] |
| O2 mean_WG | 3.5 | 2.1 |  | R:defined by [8]; A: above the level corresponding to hypoxia of 2 ml l^-1^ [39] |
| O2 mean_NBP | 3.5 | 2.1 |  | R:defined by [8]; A: above the level corresponding to hypoxia of 2 ml l^-1^; [39] |
| O2 mean_SBP | 3.5 | 2.1 |  | R:defined by [8]; A: above the level corresponding to hypoxia of 2 ml l^-1^ [39] |
| Anoxic seabed | 18.5 | 27.75 |  | R: defined by [7]; A: 50% deviance from reference [11] |
| Threat. biotop | 0 | 2 |  | R: no threatened biotopes; A: 15% of biotopes in central Baltic [11,40]) |
| Protect. biotop | 11 | 9 |  | R: all threatened (11) biotopes protected; A: 15% of theatened biotopes not protected [11] |
| Threat/Decline sp. | 0 |  | 61 | R: no threatened species; B: current state, which is defined as "Bad" by [11] |
| Protect. sp. | 61 | 52 |  | R: all threatened species protected; A: 15% of threatened species not protected [11] |
| Benthos div_BB | 12.5 | 7.5 |  | R: reference conditions defined by [11]; A: acceptable deviance from reference conditions identified by [11] |
| Benthos div_SG | 8 | 5 |  | R: reference conditions defined by [11]; A: acceptable deviance from reference conditions identified by [11] |
| Benthos div_NG | 5.5 | 3 |  | R: reference conditions defined by [11]; A: acceptable deviance from reference conditions identified by [11] |
| Benthos div_NBP | 4.9 | 3 |  | R: reference conditions defined by [11]A: acceptable deviance from reference conditions identified by [11] |
| Introd. alien sp. | 0 |  | 1 | R: no new alien invasions [40]; B: every new invasion [40] |
| Smolt prod_Moerr | 80 | 60 |  | R: smolt production capacity in Mörrumsån river [6]; A: 75% of smolt production capacity [6] |
| Smolt prod_Emån | 14 | 10.5 |  | R: smolt production capacity in Emån [6]; A: 75% of smolt production capacity [6] |
| Smolt prod_Irbe | 5 | 3.75 |  | R: smolt production capacity in Irbe river [6]; A: 75% of smolt production capacity [6] |
| Smolt prod_Venta | 14 | 10.5 |  | R: smolt production capacity in Venta river [6]; A: 75% of smolt production capacity [6] |
| Smolt prod_Saka | 7 | 5.25 |  | R: smolt production capacity in Saka river [6]; A: 75% of smolt production capacity [6] |
| Smolt prod_Uzava | 4 | 3 |  | R: smolt production capacity in Uzava river [6]; A: 75% of smolt production capacity [6] |
| Smolt prod_Barta | 4 | 3 |  | R: smolt production capacity in Barta river [6]; A: 75% of smolt production capacity [6] |
| Smolt prod_Nemunas | 148 | 111 |  | R: smolt production capacity in Nemunas river [6]; A: 75% of smolt production capacity [6] |
| Cod ssb |  | 200 | 90 | R: observed long-term level (1950s-early 1970s); earlier Bpa [41]; B: lowest level observed; suggested as Blim [41] |
| Cod rec | 300 |  | 100 | R: high level frequently observed during 1950s - early 1970s; B: lowest level observed |
| Her. ssb |  | 800 | 400 | A: suggested as a level below which extra management actions should be taken [42]; B: Bloss [43] |
| Her. rec | 20000 |  | 10000 | R: average high level observed; B: average low level observed |
| Spr. ssb |  | 400 | 200 | A: suggested as a level below which extra management actions should be taken [42]; B: lowest observed level |
| Spr. rec | 100000 |  | 15000 | R: average high level observed; B: average low level observed |
| Grey seal |  | 20000 | 4000 | A: the level in mid-2000s since when limited hunting is allowed; B: lowest observed level following historical heavy impacts of hunting and pollution |
| Ringed seal |  | 20000 | 5000 | A: the same level as for grey seals; B: 1980s level when full hunting ban was implemented; |
| cormorant_DK | 37796 |  | 2037 | R: the level reached by mid-1990s, when population stabilized after recovery; B: the low level until the early 1980s before the population started to recover |
| cormorant_MWP | 9500 |  | 705 | R: the level reached by mid-1990s, when population stabilized after recovery; B: the low level until the early 1980s before the population started to recover |
| cormorant_SH | 2450 |  | 1 | R: the level reached by mid-1990s, when population stabilized after recovery; B: the low level until the early 1980s before the population started to recover |
| Eagle | 160 |  | 80 | R: the level in early 2000s when reproduction success enabled population recovery of ca 50% [11]; B: the low level in the early 1970s due to harmful effects of pollutants |
