# Supplementary material for: Methodological Challenges in Assessing the Environmental Status of a Marine Ecosystem: Case Study of the Baltic Sea
Source: PLoS One. 2011 Apr 29;6(4):e19231. doi: 10.1371/journal.pone.0019231 (PMC3084783; doi:10.1371/journal.pone.0019231)
Supplement: Table S3 — Structure for aggregating state indicators. Hierarchical structure for aggregating state indicators (shown by their acronyms; see Table S1 for indicator descriptions) into Objectives and Goals via up to three intermediate steps (Steps 1–3). (DOC) [file pone.0019231.s003.doc]

| **Goal** | **Objective** | **Step 3** | **Step 2** | **Step 1** | **Indicator** |
| --- | --- | --- | --- | --- | --- |
| Biodiversity | Habitat |  |  |  | Anoxic seabed |
|  |  |  |  |  | Threat. biotop |
|  |  |  |  |  | Protect. biotop |
|  |  | Threat/Protect. sp. |  |  | Threat/Decline sp. |
|  |  |  |  |  | Protect. sp. |
|  | Communities | Benthos div. |  |  | Benthos div_BB |
|  |  |  |  |  | Benthos div_SG |
|  |  |  |  |  | Benthos div_NG |
|  |  |  |  |  | Benthos div_NBP |
|  |  | Introd. alien sp. |  |  | Introd. alien sp. |
|  | Populations | Fish populations | Salmon |  | Smolt prod_Moerr |
|  |  |  |  |  | Smolt prod_Emån |
|  |  |  |  |  | Smolt prod_Irbe |
|  |  |  |  |  | Smolt prod_Venta |
|  |  |  |  |  | Smolt prod_Saka |
|  |  |  |  |  | Smolt prod_Uzava |
|  |  |  |  |  | Smolt prod_Barta |
|  |  |  |  |  | Smolt prod_Nemunas |
|  |  |  | Cod |  | Cod ssb |
|  |  |  |  |  | Cod rec |
|  |  |  | Herring |  | Her. ssb |
|  |  |  |  |  | Her. rec |
|  |  |  | Sprat |  | Spr. ssb |
|  |  |  |  |  | Spr. rec |
|  |  | Marine mammals |  |  | Grey seal |
|  |  |  |  |  | Ringed seal |
|  |  | Seabirds | Cormorant |  | cormorant_DK |
|  |  |  |  |  | cormorant_MWP |
|  |  |  |  |  | cormorant_SH |
|  |  |  | Eagle |  | Eagle |
| Eutrophication | Nutrients | Open | N open sea |  | DIN open |
|  |  |  |  |  | TN open |
|  |  |  | P open sea |  | DIP open |
|  |  |  |  |  | TP open |
|  |  | Coast | N coast |  | DIN coast |
|  |  |  |  |  | TN coast |
|  |  |  | P coast |  | DIP coast |
|  |  |  |  |  | TP coast |
|  | Clear water | Secchi |  |  | Secchi_WG |
|  |  |  |  |  | Secchi_EG |
|  |  |  |  |  | Secchi_NBP |
|  |  |  |  |  | Secchi_BB |
|  | Algal blooms | Chl a |  |  | Chl a open |
|  |  |  |  |  | Chl a coast |
|  |  | Cyano ind. |  |  | Cyano ind. |
|  | Biota distribution | Fucus |  |  | Fucus mean |
|  |  |  |  |  | Fucus max |
|  | Oxygen level | O2 low |  |  | O2 low_EG |
|  |  |  |  |  | O2 low_WG |
|  |  |  |  |  | O2 low_NBP |
|  |  |  |  |  | O2 low_SBP |
|  |  | O2 mean |  |  | O2 mean_EG |
|  |  |  |  |  | O2 mean_WG |
|  |  |  |  |  | O2 mean_NBP |
|  |  |  |  |  | O2 mean_SBP |
| Hazardous substances | Residuals | H.metals |  |  | Hg Guill. |
|  |  |  | Chlorin | DDT | sDDT Guill. |
|  |  |  |  |  | DDE Eagle |
|  |  |  |  | PCB | sPCB Guill. |
|  |  |  |  |  | PCB Eagle |
|  |  | Persist. org. pollut. |  | HCH | b_HCH Guill. |
|  |  |  |  |  | HCB Guill. |
|  |  |  | Dioxin |  | TCDD Guill. |
|  |  |  | Bromin |  | BDE_47 Guill. |
|  |  |  |  |  | HBCD Guill. |
|  |  |  | Flouorin |  | PFOS Guill. |
|  | Fish safe to eat | Cod | Cod: H.met. |  | Cd Cod |
|  |  |  |  |  | Cu Cod |
|  |  |  |  |  | Hg Cod |
|  |  |  |  |  | Pb Cod |
|  |  |  |  |  | Zn Cod |
|  |  |  | Cod: Chlorin |  | sDDT cod |
|  |  | Herring | Her: H.met. | Cd_her | Cd Her_Ut |
|  |  |  |  |  | Cd Her_Ls |
|  |  |  |  | Cu_her | Cu Her_Ut |
|  |  |  |  |  | Cu Her_Ls |
|  |  |  |  | Hg_her | Hg Her_Ut |
|  |  |  |  |  | Hg Her_Ls |
|  |  |  |  | Pb_her | Pb Her_Ut |
|  |  |  |  |  | Pb Her_Ls |
|  |  |  |  | Zn_her | Zn Her_Ut |
|  |  |  |  |  | Zn Her_Ls |
|  |  |  | Her: Chlorin | DDT_her | sDDT Her_Ut |
|  |  |  |  |  | sDDT Her_Ls |
|  |  |  |  | PCB_her | sPCB Her_Ut |
|  |  |  |  |  | sPCB Her_Ls |
|  |  |  | Her: Dioxin |  | TCDD Her_SB |
|  |  |  |  |  | TCDD Her_EB |
|  |  |  |  |  | TCDD Her_POL |
|  |  |  |  |  | TCDD Her_LAT |
|  |  |  |  |  | TCDD Her_SG |
|  |  |  | Her: Bromin |  | HBCD her_Ut |
|  |  |  |  |  | HBCD her_Ls |
|  |  | Salmon | Salmon: Dioxin |  | TCDD salm. |
|  | Healthy wildlife | Reproduction | Eagle reprod. |  | Eagle repr. suc_MWP |
|  |  |  |  |  | Eagle repr. suc_BP |
|  |  |  |  |  | Eagle br. size_BP |
|  |  |  | Seal reprod. |  | Seal ut. obstr. |
|  |  |  |  |  | Seal ut. leiom. |
|  |  |  |  |  | Seal pct pregn. |
|  |  | Disease |  |  | Salmon M74 |
|  |  |  |  |  | Seal int. ulcers |
|  | Radioactivity | Water |  |  | Cs_137_BPN |
|  |  |  |  |  | Cs_137_BPS |
|  |  |  |  |  | Cs_137_BPM |
|  |  | Biota |  |  | Cs_137 Her_BPN |
|  |  |  |  |  | Cs_137 Her_BPS |
|  |  |  |  |  | Cs_137 Flatf_EB |
|  |  |  |  |  | Sr_90 pike_EB |
|  |  |  |  |  | Cs_137 Fuc_WB |
