# Supplementary material for: Methodological Challenges in Assessing the Environmental Status of a Marine Ecosystem: Case Study of the Baltic Sea
Source: PLoS One. 2011 Apr 29;6(4):e19231. doi: 10.1371/journal.pone.0019231 (PMC3084783; doi:10.1371/journal.pone.0019231)
Supplement: Table S4 — Structure for aggregating pressure indicators. Hierarchical structure for aggregating pressure indicators (shown by their acronyms; see Table S1 for indicator descriptions) by sources of pressure via an intermediate step (Step 1), where relevant. (DOC) [file pone.0019231.s004.doc]

# Structure for aggregating pressure indicators

Table S4. Hierarchical structure for aggregating pressure indicators (shown by their acronyms; see Table S1 for indicator descriptions) by sources of pressure via an intermediate step (Step 1), where relevant.

| **Source of pressure** | **Step 1** | **Indicator** |
| --- | --- | --- |
| Input of heavy metals and dioxins |  | Hg inp. air |
|  |  | Cd inp. air |
|  |  | Pb inp. air |
|  |  | Hg inp. water |
|  |  | Cd inp. water |
|  |  | Pb inp. water |
|  |  | PCDD/Fs inp. air |
| Discharge of radionuclides | Cs-137 | Cs_137_Oskh |
|  |  | Cs_137_Studs |
|  | Sr-90 | Sr_90_Oskh |
|  |  | Sr_90_Studs |
|  | Co-60 | Co_60_Oskh |
|  |  | Co_60_Studs |
| Nutrient input |  | Direct N inp. |
|  |  | N oxid. air |
|  |  | N red. air |
|  |  | Direct P inp. |
| Shipping |  | Illeg. oil disch. |
|  |  | Poll. accid. |
|  |  | Vessels Kiel Can. |
|  |  | Cargo ton. Kiel Can. |
|  |  | Vessel traffic |
| Fishing | Cod expl. | Cod expl. ad. |
|  |  | Cod expl. rec. |
|  | Her. expl. | Her. expl. |
|  | Spr. expl. | Spr. expl. |
|  | Seal by-catch | Seal by-catch |
| Hunting/Shooting | Seal hunt. | Seal hunt. |
|  | Cormorant shoot. | Corm. shoot. DK |
|  |  | Corm. shoot. MWP |
|  |  | Corm. shoot. SH |
|  |  | Corm. shoot. EST |
