# Supplementary material for: Methodological Challenges in Assessing the Environmental Status of a Marine Ecosystem: Case Study of the Baltic Sea
Source: PLoS One. 2011 Apr 29;6(4):e19231. doi: 10.1371/journal.pone.0019231 (PMC3084783; doi:10.1371/journal.pone.0019231)
Supplement: Text S1 — List of references cited in Tables S1, S2, S3, S4. (DOC) [file pone.0019231.s005.doc]

# References

1. Bignert A, Nyberg E, Asplund L, Eriksson U, Wilander A, et al. (2007) Metaller och organiska miljögifter i marin biota, trend- och områdesövervakning. Sakrapport. Stockholm: Swedish Museum of Natural History. 129 p.
2. Bignert A, Danielsson S, Nyberg E, Asplund L, Eriksson U, et al. (2009) Övervakning av metaller och organiska miljögifter i marin biota. Sakrapport. Stockholm: Swedish Museum of Natural History. 154 p.
3. Helander B, Bignert A, Herrmann C (2009) Predatory bird health - white-tailed sea eagle. HELCOM Indicator Fact Sheets 2009. Online. [Viewed 01 March 2010], <http://www.helcom.fi/environment2/ifs/en_GB/cover/>.
4. HELCOM (2004) Dioxins in the Baltic Sea. Helsinki: HELCOM. 20 p.
5. Bäcklin B-M, Moraeus C, Kunnasranta M, Isomursu M (2009) Health Assessment in the Baltic grey seal (Halichoerus grypus). HELCOM Indicator Fact Sheets 2009. Online. [Viewed 01 March 2010], <http://www.helcom.fi/environment2/ifs/en_GB/cover/>.
6. ICES (2009) Report of the Baltic Salmon and Trout Assessment Working Group (WGBAST), 24-31 March 2009, Oulu, Finland. ICES CM 2009/ACOM:05. Copenhagen: ICES. 276 p.
7. HELCOM (2009) Radioactivity in the Baltic Sea, 1999-2006. HELCOM thematic assessment. Baltic Sea Environmental Proceedings 117. Helsinki: HELCOM. 64 p.
8. HELCOM (2009) Eutrophication in the Baltic Sea – An integrated thematic assessment of the effects of nutrient enrichment and eutrophication in the Baltic Sea region. Baltic Sea Environmental Proceedings 115B. Helsinki: HELCOM. 152 p.
9. Fleming-Lehtinen V, Kaartokallio H (2009) Water transparency in the Baltic Sea between 1903 and 2009. HELCOM Indicator Fact Sheets 2009. Online. [Viewed 01 March 2010], <http://www.helcom.fi/environment2/ifs/en_GB/cover/>.
10. Kaitala S, Hällfors S (2008) Cyanobacteria bloom index. HELCOM Indicator Fact Sheets 2008. Online. [Viewed 01 March 2010], <http://www.helcom.fi/environment2/ifs/en_GB/cover/>.
11. HELCOM (2009) Biodiversity in the Baltic Sea – An integrated thematic assessment on biodiversity and nature conservation in the Baltic Sea. Baltic Sea Environmental Proceedings 116B. Helsinki: HELCOM. 192 p.
12. HELCOM (2007) HELCOM lists of threatened and/or declining species and biotopes/habitats in the Baltic Sea area. Baltic Sea Environmental Proceedings 113. Helsinki: HELCOM. 18 p.
13. HELCOM (2010) Status and cocherence of of the network of Baltic Sea Protected Areas including Natura 2000. Document 4/4: Agenda Item 4 Contributions to the 2010 HELCOM Ministerial Meeting by HELCOM HABITAT. Nature Protection and Biodiversity Group Twelfth Meeting Tallinn, Estonia, 17-19 February 2010. Helsinki: HELCOM. 157 p.
14. Baltic Sea Alien Species Database (2009) Online. [Viewed 27 January 2010], <http://www.ku.lt/nemo/mainnemo.htm>.
15. ICES (2009) Report of the Baltic Fisheries Assessment Working Group (WGBFAS), 22-28 April 2009, ICES Headquarters, Copenhagen. ICES CM 2009\ACOM:07. Copenhagen: ICES. 635 p.
16. Harding KC, Härkönen TJ (1999) Development in the Baltic grey seal (*Halichoerus grypus*) and ringed seal (*Phoca hispida*) populations during the 20th century. Ambio 28: 619–627
17. ICES (2007) Report of the Working Group on Marine Mammal Ecology (WGMME), 27–30 March 2007, Vilm, Germany. ICES CM 2007/ACE:03. Copenhagen: ICES. 61 p.
18. Aro E, ed (2008) Seal and Society. Tema Nord. 585 p.
19. Herrmann C, Bregnballe T, Larsson K, Ojaste I, Lilleleht V (2009). Population Development of Baltic Bird Species: Great Cormorant (*Phalacrocorax carbosinensis*). HELCOM Indicator Fact Sheets 2009. Online. [Viewed 27 January 2010], <http://www.helcom.fi/environment2/ifs/en_GB/cover/>.
20. Herrmann C, Krone O, Stjernberg T, Helander B (2009) Population Development of Baltic Bird Specie: White-tailed Sea Eagle (Haliaeetus albicilla). HELCOM Indicator Fact Sheets 2009. Online. [Viewed 01 March 2010], <http://www.helcom.fi/environment2/ifs/en_GB/cover/>
21. Gusev A (2009) Atmospheric deposition of heavy metals on the Baltic Sea. HELCOM Indicator Fact Sheets 2009. Online. [Viewed 01 March 2010], <http://www.helcom.fi/environment2/ifs/en_GB/cover/>.
22. Knuutila S, ed (2009) Waterborne inputs of heavy metals to the Baltic Sea. HELCOM Indicator Fact Sheets 2009. Online. [Viewed 01 March 2010], <http://www.helcom.fi/environment2/ifs/en_GB/cover/>.
23. Gusev A (2009) Atmospheric depositions of PCDD/Fs on the Baltic Sea. HELCOM Indicator Fact Sheets 2009. Online. [Viewed 01 March 2010], <http://www.helcom.fi/environment2/ifs/en_GB/cover/>.
24. Vartti V-P (2009) Liquid discharges of Cs-137, Sr-90 and Co-60 into the Baltic Sea from local nuclear installations. HELCOM Indicator Fact Sheets 2009. Online. [Viewed 01 March 2010], <http://www.helcom.fi/environment2/ifs/en_GB/cover/>.
25. Bartnicki J (2009) Atmospheric nitrogen deposition to the Baltic Sea during 1995-2007. HELCOM Indicator Fact Sheets 2009. Online. [Viewed 01 March 2010], <http://www.helcom.fi/environment2/ifs/en_GB/cover/>.
26. HELCOM (2009) Illegal discharges of oil in the Baltic Sea during 2008. HELCOM Indicator Fact Sheets. Online. [Viewed 01 March 2010], <http://www.helcom.fi/environment2/ifs/en_GB/cover/>.
27. HELCOM (2008) Report on shipping accidents in the Baltic Sea area for the year 2008. Helsinki: HELCOM. 30 p.
28. Gollasch S, Rosenthal H (2006) The Kiel Canal. In: Gollasch S, Galil BS, Cohen AN , eds. Bridging divides: Maritime canals as invasion corridors. Springer pp. 5-90.
29. HELCOM (2008). HELCOM Automatic Identification System. Online. [Viewed 01 March 2010]. <http://www.helcom.fi/shipping/navigation/en_GB/navigation/>
30. Commission of the European Communities (2001). EU commission decision 466/2001/EC of 8 March 2001 setting maximum levels for certain contaminants in foodstuffs. Official Journal of the European Communities L77/1 of 16/03/2001.
31. Odsjö T, Olsson M (1989) Miljöprovbanken-ny institution i miljövårdens tjänst. In: Engström K, ed. Naturen berättar. Swedish Museum of Natural History.
32. Commission of the European Communities 2008. Commission regulation (EC) No 149/2008 of 29 January 2008 amending Regulation (EC) No 396/2005 of the European Parliament and of the Council by establishing Annexes II, III and IV setting maximum residue levels for products covered by Annex I thereto. Official Journal of the European Union L58/1 of 01/03/2008.
33. Commission of the European Communities (1999). EU commission decision 1999/788/EC of 3 December 1999 on protective measures with regard to contamination by dioxins of certain products of porcine and poultry origin intended for human or animal consumption. Official Journal of the European Union L 310/62 of 04/12/1999.
34. Commission of the European Communities (2006) EU commission decision 1881/2006EC of 19 December 2006 on setting maximum levels for certain contaminants in foodstuffs. Official Journal of the European Union L 364/5 of 20/12/2006.
35. HELCOM (2006) Development of tools for assessment of eutrophication in the Baltic Sea. Baltic Sea Environmental Proceedings 104. Helsinki: HELCOM. 64 p.
36. Fleming-Lehtinen V, Kaartokallio H, Olsonen R (2008) Water transparency in the Baltic Sea between 1903 and 2008. HELCOM Indicator Fact Sheets 2008. Online. [Viewed 01 March 2010], <http://www.helcom.fi/environment2/ifs/en_GB/cover/>.
37. Kautsky N, Kautsky H, Kautsky U, Waern M (1986) Decreased depth penetration of *Fucus vesiculosus* (L.) since the 1940s indicates eutrophication of the Baltic Sea. Marine Ecology Progress Series 28: 1–8.
38. Finni T, Kononen K, Olsonen R, Wallström K (2001) The history of cyanobacterial blooms in the Baltic Sea. Ambio 30: 172–178.
39. Conley DJ, Björk S, Bonsdorff E, Carstensen J, Destouni G et al. (2009) Hypoxia-related processes in the Baltic Sea. Environmental Science and Technology 43: 3412–3420.
40. HELCOM (2007) Baltic Sea Action Plan. Helsinki: HELCOM. 102 p.
41. ICES (2007) Report of the Workshop on Limit and Target Reference Points. ICES CM2007/ACFM:05. Copenhagen: ICES.
42. ICES (2009). Report of the Workshop on Multi-annual management of Pelagic Fish Stocks in the Baltic. ICES CM 2009/ACOM:38. Copenhagen: ICES, Copenhagen.
43. ICES (2008) Report of the Baltic Fisheries Assessment Working Group (WGBFAS). ICES CM 2008/ACOM:06. Copenhagen: ICES. 700 p.
